# Effects of pH and Nutrients (Nitrogen) on Growth and Toxin Profile of the Ciguatera-Causing Dinoflagellate *Gambierdiscus polynesiensis* (Dinophyceae)

**DOI:** 10.3390/toxins12120767

**Published:** 2020-12-04

**Authors:** Sébastien Longo, Manoëlla Sibat, Hélène Taiana Darius, Philipp Hess, Mireille Chinain

**Affiliations:** 1 Laboratory of Marine Biotoxins, Institut Louis Malardé-UMR241 EIO (IFREMER, ILM, IRD, UPF), 98713 Papeete, Tahiti, French Polynesia; tdarius@ilm.pf (H.T.D.); mchinain@ilm.pf (M.C.); 2 Phycotoxins Laboratory, IFREMER, Rue de I’lle d’Yeu, 44311 Nantes, France; Manoella.Sibat@ifremer.fr (M.S.); Philipp.Hess@ifremer.fr (P.H.)

**Keywords:** *Gambierdiscus polynesiensis*, French Polynesia, ciguatera, ciguatoxins, LC-MS/MS, toxin profile, nitrate, urea, culture medium acidification

## Abstract

Ciguatera poisoning is a foodborne disease caused by the consumption of seafood contaminated with ciguatoxins (CTXs) produced by dinoflagellates in the genera *Gambierdiscus* and *Fukuyoa*. Ciguatera outbreaks are expected to increase worldwide with global change, in particular as a function of its main drivers, including changes in sea surface temperature, acidification, and coastal eutrophication. In French Polynesia, *G. polynesiensis* is regarded as the dominant source of CTXs entering the food web. The effects of pH (8.4, 8.2, and 7.9), Nitrogen:Phosphorus ratios (24N:1P vs. 48N:1P), and nitrogen source (nitrates vs. urea) on growth rate, biomass, CTX levels, and profiles were examined in four clones of *G. polynesiensis* at different culture age (D10, D21, and D30). Results highlight a decrease in growth rate and cellular biomass at low pH when urea is used as a N source. No significant effect of pH, N:P ratio, and N source on the overall CTX content was observed. Up to ten distinct analogs of Pacific ciguatoxins (P-CTXs) could be detected by liquid chromatography-tandem mass spectrometry (LC-MS/MS) in clone NHA4 grown in urea, at D21. Amounts of more oxidized P-CTX analogs also increased under the lowest pH condition. These data provide interesting leads for the custom production of CTX standards.

## 1. Introduction

Ciguatera poisoning (CP) is the most prevalent, phycotoxin-related seafood intoxication affecting mainly populations living in tropical and subtropical areas of the world. CP cases occur after the consumption of seafood contaminated with toxins known as ciguatoxins (CTXs) that are produced by dinoflagellates in the genera *Gambierdiscus* and *Fukuyoa*. Ciguatera toxins enter marine food webs through grazing by herbivores and detritivores and are further accumulated through predation and bio-transformed in carnivores [1,2]. Overall, eighteen species of *Gambierdiscus* are now recognized worldwide [3,4,5,6,7,8,9,10,11,12,13,14]. The phylogeny of these species based on large subunit ribosomal ribonucleic acid (LSU rDNA) D8–D10 region sequences is provided by Kretzschmar et al. [11]. Ciguatoxin production has been shown by the neuroblastoma cell-based assay (CBA-N2a) to vary considerably according to species [15,16] but, interestingly, has only been confirmed by liquid chromatography-tandem mass spectrometry (LC-MS/MS) in *G. polynesiensis* since the genus *Gambierdiscus* has been enlarged from *G. toxicus* to other species [3,17].

Numerous field observations linking the effects of climate change to CP events can be found in the literature [18,19,20,21,22,23,24]. In particular, cyclical weather patterns such as El Niño, associated with unusual warming of ocean waters in the Pacific, have resulted in spikes of ciguatera [25], consistent with observations by Gingold et al. [26], who found an association between CP incidence and warmer sea surface temperatures in the Caribbean basin. Many laboratory studies have also investigated *Gambierdiscus* and *Fukuyoa* spp. growth responses and/or toxin production under varying environmental conditions, including temperature, salinity, and irradiance (for reviews, see References [27] and [28], and references therein). Results confirm that differences among strains exist not only across but also within species [15,29,30,31,32,33]. All these observations substantiate the idea that ocean warming as a result of climate change may increase the global distribution of CTX-producing dinoflagellates and, hence, CP incidence worldwide. However, the influence of other environmental drivers commonly associated with global change, such as ocean acidification and increasing eutrophication of coastal marine ecosystems, also deserve to be investigated.

Oceans serve as one of the largest carbon reserves on earth, absorbing nearly one third of all carbon dioxide emissions [34]. Increasing amounts of CO_2_ diffusing from the atmosphere into the oceans results, among others, in ocean acidification [35], currently estimated as a decrease in seawater pH by 0.11 units since the 1870s [36], with the current pH being 8.06 on average [37]. Following the fifth Intergovernmental Panel on Climate Change report, forecasts predict an additional decrease in ocean pH from 0.07 (Representative Concentration Pathway 2.6; RCP 2.6) to 0.33 units (RCP 8.5) by 2100 [37,38]. Furthermore, over the past decades, there have also been significant increases in marine eutrophication globally [39]. Still, eutrophication should be considered a localized phenomenon and, in some regions, improvements have shown to lead to reduction of harmful algal blooms (HABs), e.g., in the Japanese Inland Sea [40]. Nitrogen (N) and phosphorus (P), two major drivers of marine eutrophication, are commonly regarded as key limiting nutrients in most aquatic ecosystems [41]. Eutrophication episodes can lead to hypoxia and anoxia, reduced water quality, alteration of food web structure, habitat degradation, loss of biodiversity, and even harmful algal bloom (HAB) events [39,42,43,44], which in turn may result in health risks and economic losses, including losses of fish and wildlife production and losses of recreational amenities [45,46]. By way of example, French Polynesian lagoons are well-known oligotrophic ecosystems characterized by low concentrations of dissolved mineral nitrogen [47,48,49]. However, more recently, intensified agriculture and aquaculture (e.g., pearl-farming) practices, as well as increasing development of coastal activities, have caused major changes in local environments via the release of nutrient-rich inputs [44,50,51].

The influence nutrients and pH have on the ecophysiology and toxicity of ciguatera-causing dinoflagellates is still poorly documented. To our knowledge, no published study has currently investigated the effects of pH on the growth and toxin production of *Gambierdiscus* and *Fukuyoa* spp. However, several studies have shown the effect of pH on various HAB species (Reference [47] and references therein), such as *Alexandrium* and *Pseudo-Nitzschia* spp. [48,49,50,51,52,53,54,55,56,57]. Likewise, few field studies have examined the correlation between *Gambierdiscus* cell densities and nutrient levels in various ciguatera-prone areas of the Pacific (French Polynesia, Hawaii) [57,58,59], Caribbean (British and U.S. Virgin Islands) [60,61,62], and Indian Ocean (Mauritius) [63]. While some of these studies were limited by non-detectable nutrient concentrations [63], others gave contradictory results [57,58,59,60,61,62]. There is also limited data on the role of nutrients in stimulating *Gambierdiscus* and *Fukuyoa* spp. toxin production [32,64,65,66,67]. These studies, which examined the effects of both nutrient levels and various nitrogen sources (i.e., nitrate, ammonium, urea), showed that species matters, as results suggest that the various species of *Gambierdiscus* may have different nutrient physiologies and uptake kinetics.

In French Polynesia, *G. polynesiensis* is regarded as the dominant source of CTXs entering the food web [68]. A recent study conducted on four *G. polynesiensis* isolates originating from distinct geographic locations in French Polynesia, showed that these strains produce at least nine distinct P-CTX analogs in culture, namely P-CTX4B, P-CTX4A (52-epi-P-CTX4B), P-CTX3C, P-CTX3B (49-epi-P-CTX3C), 2-hydroxy-P-CTX3C, M-seco-P-CTX3C, and three P-CTX3B/C isomers not fully characterized yet, and confirmed that clonal variation in toxin production exists in this species [33].

As a follow-up study, the present work aimed at assessing the in vitro growth and toxin production in *G. polynesiensis* at different growth phases, when cultured under various combinations of pH (8.4, 8.2, and 7.9) and Nitrogen:Phosphorus (N:P) ratios (24N:1P vs. 48N:1P), while using two distinct N sources, i.e., nitrate vs. urea. Variations in both overall cellular CTX concentrations and toxin profiles were characterized by LC-MS/MS. How these data can help promote the custom production of algal CTX standards currently needed for the implementation of CP risk and management programs is further discussed.

## 2. Results

### 2.1. Effects of Culture Age, pH, Nitrogen Source, and N:P Ratio on G. polynesiensis Growth Rate and Biomass

The mean growth rates exhibited by each *G. polynesiensis* strain 10 days (D10) and 21 days (D21) post-inoculation, under three different pH and two N:P ratios using either nitrate or urea as N source, are presented in Table 1.

In nitrate growth experiments, all strains displayed a typical growth pattern at pH = 8.4 and 8.2, characterized by a maximum growth rate of 0.2 division day^−1^ (div day^−1^) on average at the exponential growth phase (from D0 to D10) and a mean growth rate of 0.1 div day^−1^ at the stationary phase (from D10 to D21), whatever the N:P ratio tested. At pH = 7.9, although a similar growth rate around 0.2 div day^−1^ was reached at the exponential growth phase, cultures rapidly declined after 10 days contrarily to cultures maintained at pH 8.4 or 8.2. Overall, for *G. polynesiensis* considered as a whole, statistical analyses revealed no significant effect of pH or N:P ratio on the growth rate in nitrate-based culture media (data not shown).

In urea growth experiments, a different growth pattern was demonstrated in all four strains as compared to nitrate conditions (Table 1). From D0 to D10, growth rates never exceeded 0.14 div day^−1^ (clone RIK7, 48N:1P) at pH = 8.4, for the two N:P ratios tested. At pH = 8.2, strains showed a different growth response characterized by a latency phase from D0 to D10, an exponential phase from D10 to D21 with a maximum growth rate of 0.2 div day^−1^ (RIK7, 48N:1P), followed by a stationary phase from D21 to D30 for all strains (data not shown). At pH = 7.9, a similar growth trend was observed, although growth rates were generally lower than at pH = 8.2 (Table 1).

The cell biomass reached after 30 days of culture are presented in Table 2. It can be noted that data significantly differed between nitrate and urea supply, most notably at 24N:1P ratio (Table 2), with final cell yields in nitrate being 3-fold higher on average than those obtained in urea. At a given pH, no N:P ratio effect on cell biomass was noticed when strains were grown in nitrate, whereas cell yields obtained for all strains at 48N:1P were almost 2-fold higher on average than at 24N:1P in urea. For a given N source, cell yields were also lower at pH = 7.9 than at pH = 8.4 at the two N:P ratios tested. Of note, in nitrate conditions, the lowest cell yields were generally observed in the RAI-1 strain.

### 2.2. Diversity of P-CTX Analogs in G. polynesiensis

**Figure 1:**
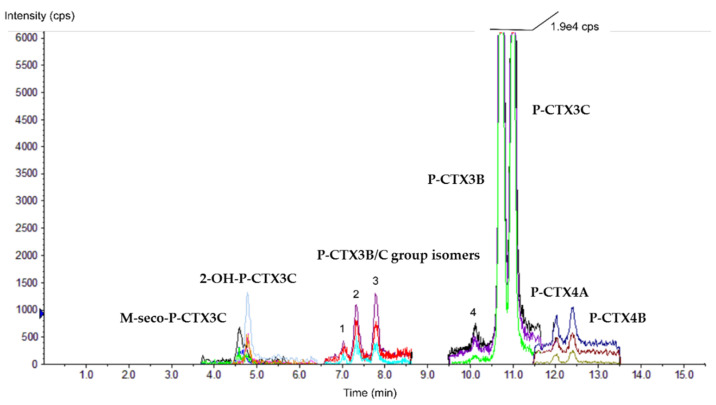
Chromatogram of scheduled Multiple Reaction Monitoring (MRM) LC-MS/MS showing the different P-CTX analogs detected in crude methanol extracts of *G. polynesiensis* strain NHA4 cultivated during 21 days in urea 48N:1P at pH = 7.9. The different colors correspond to the different MRM transitions (*m*/*z*) selected for the analysis method, scanning three transitions for each P-CTX analogs (see Supplementary Table S1 for further information about *m*/*z* transitions selected).

The LC-MS/MS analyses performed on crude methanol extracts of all four clones revealed that depending on the strain and culture conditions applied, the number of detectable P-CTX analogs in cultures of *G. polynesiensis* could range from four up to ten analogs consisting of: (i) two major compounds, P-CTX3B and P-CTX3C, and (ii) eight minor CTXs analogs: two non-polar analogs from the P-CTX1B group, i.e., P-CTX4A and P-CTX4B, two oxidized forms of P-CTX3C, i.e., 2-OH-P-CTX3C and M-seco-P-CTX3C, and four hitherto undescribed isomers belonging to the P-CTX3B/C group, referred to as P-CTX3B/C group isomers 1, 2, 3, and 4 (Figure 1).

Of note, only P-CTX3B/C group isomers 2 and 3 were produced at quantifiable amounts, whereas P-CTX3B/C isomers 1 and 4 were consistently present at levels below the limit of quantification (LOQ = 10 ng mL^−1^) and, therefore, are not represented in the following figures and tables.

Quantitation of the different P-CTXs analogs was carried out using P-CTX3C, the only commercially available reference material. Assuming equal molar response and applying the same LOD and LOQ, the estimated concentrations were calculated against the P-CTX3C calibration curve and expressed in equivalent P-CTX3C. The sum of the estimated concentrations of all analogs allowed estimation of the overall cellular toxin content (expressed in pg P-CTX3C eq cell^−1^).

### 2.3. Effects of Strain, Culture Age, Nitrogen, and pH on the Overall P-CTX Cell Content in G. polynesiensis

**Figure 2:**
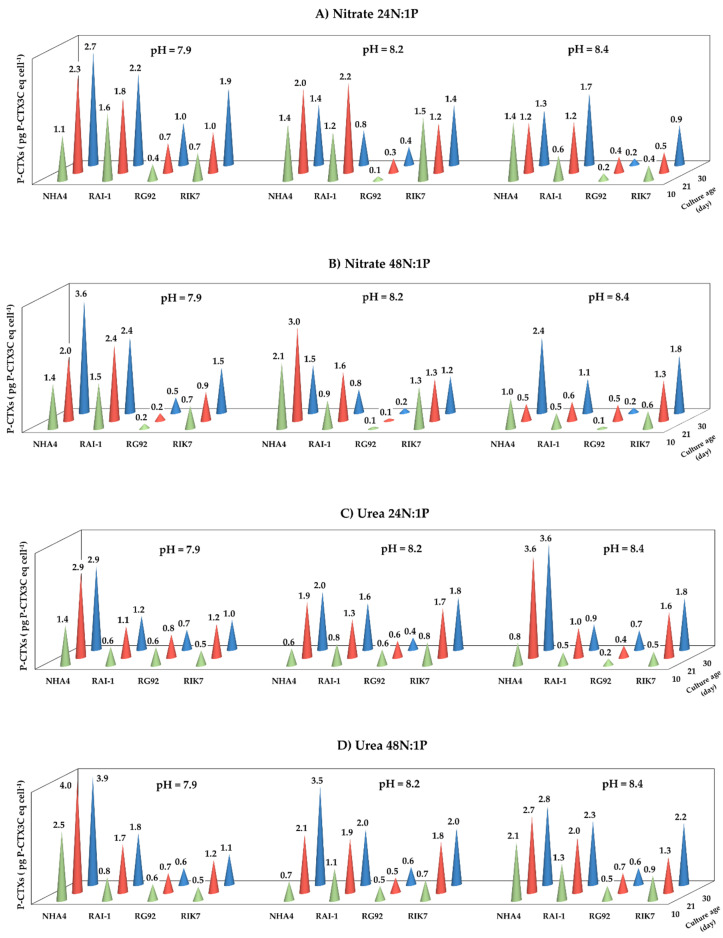
Mean overall P-CTX content (expressed in pg P-CTX3C eq cell^−1^) measured by LC-MS/MS at three distinct culture ages (D10, D21, and D30) in four *G. polynesiensis* strains (NHA4, RAI-1, RG92, and RIK7) when grown at three different pH (7.9, 8.2, and 8.9), in nitrate: 24N:1P (**A**) and 48N:1P (**B**) vs. urea: 24N:1P (**C**) and 48N:1P (**D**). Data represent the mean P-CTX content in each strain tested in two technical replicates.

The overall P-CTX cell content in the four strains ranged from 0.1 to 4.0 pg P-CTX3C eq cell^−1^, depending on culture age, strain, and culture conditions (Figure 2). Of note, the P-CTX content in NHA4 (i.e., 2.12 ± 0.99 pg P-CTX3C eq cell^−1^, on average) was significantly higher than the one measured in the three other clones (*p*-values ranged from <0.0001 to 0.004). Conversely, RG92 showed the lowest P-CTX content with an average of 0.44 ± 0.24 pg P-CTX3C eq cell^−1^ (Figure 2). Also, P-CTX contents measured at D10 were significantly lower than at D21 and D30 (*p*-value < 0.05). In contrast, the pH, N source, or N:P ratio had no significant effects on the overall cellular CTX content of *G. polynesiensis* strains.

### 2.4. Effects of Strain, Culture Age, Nitrogen, and pH on G. polynesiensis Toxin Profiles

#### 2.4.1. Clonal Variations in P-CTX Profiles

**Figure 3:**
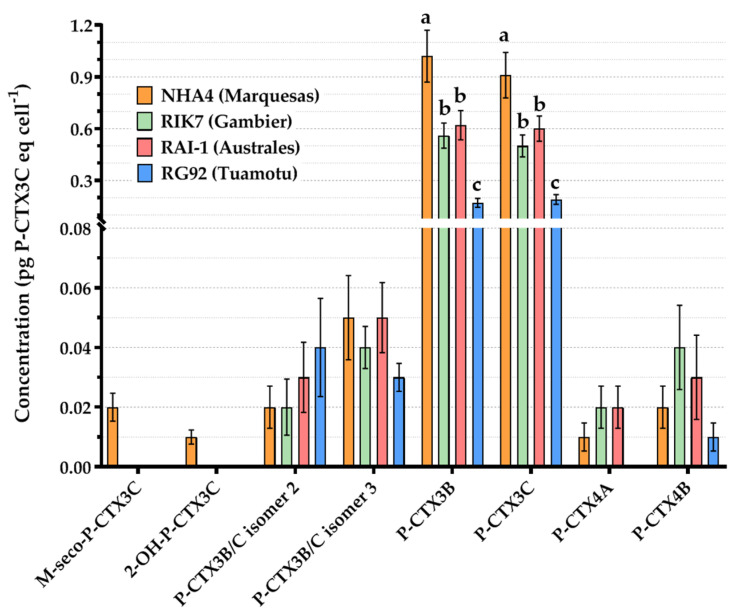
Mean cellular concentrations expressed in pg P-CTX3C equivalent cell^−1^ (eq cell^-1^) of each P-CTX analog measured by LC-MS/MS in four *G. polynesiensis* strains (NHA4, RIK7, RAI-1, and RG92). Data represent the mean ± 95% confidence interval (CI) of n = 72 samples (two technical replicates per strain when grown under three pH conditions and two different N sources tested at two N:P ratios, at three culture ages). The letters a, b and c shown on P-CTX3B and -3C bars indicate statistical differences observed between clones based on pairwise comparisons.

While RAI-1 (Australes strain) and RIK7 (Gambier strain) had similar toxin profiles composed of six analogs in all experiments (Figure 3), two additional analogs, i.e., 2-OH-P-CTX3C and M-seco-P-CTX3C, were quantified in NHA4 (Marquesas strain) cultures, whereas P-CTX4A analog was not quantified in RG92 (Tuamotu strain). Figure 3 shows that the amounts of P-CTX3B and P-CTX3C were significantly higher in NHA4 than in the three other clones (*p*-value < 0.0001), and significantly lower in RG92 than in other clones (*p*-value < 0.0001), based on pairwise comparisons.

#### 2.4.2. Effects of Culture Age on *G. polynesiensis* P-CTX Concentrations

**Figure 4:**
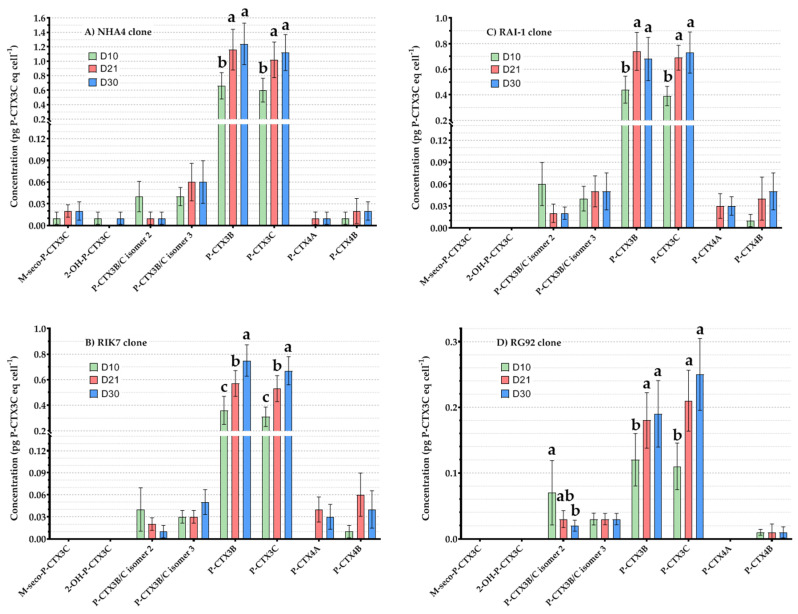
Mean cellular concentrations (expressed in pg P-CTX3C eq cell^−1^) of P-CTX analogs measured by LC-MS/MS in four *G. polynesiensis* clones at three different culture ages (D10, D21, and D30): (**A**) NHA4, (**B**) RIK7, (**C**) RAI-1, and (**D**) RG92. Data represent the mean ± 95% CI of n = 24 samples (two technical replicates per strain when grown under three pH conditions and two different N sources tested at two N:P ratios). Letters indicate statistical differences observed between samples based on pairwise comparisons.

The concentrations of the different P-CTX analogs detected in each of the four clones at different culture ages, i.e., D10, D21, and D30, are presented in Figure 4. Overall, only five analogs could be quantified at D10 vs. up to eight analogs at D30 depending on the strain considered (Figure 4). Moreover, a progressive increase in P-CTX3B and -3C concentrations, the two major toxins produced by *G. polynesiensis* strains, was noticed in RIK7 from D10 to D30 (Figure 4B), whereas for the three other clones, no significant differences were observed between D21 and D30 for these two compounds (Figure 4A,C,D). Interestingly, while P-CTX3B/C isomer 2 significantly decreased between D10 and D30 in strain RG92 (Figure 4D), P-CTX4A and -4B concentrations increased in the three other strains (Figure 4A–C).

#### 2.4.3. Effects of N Source and N:P Ratio on *G. polynesiensis* P-CTXs Concentrations

**Figure 5:**
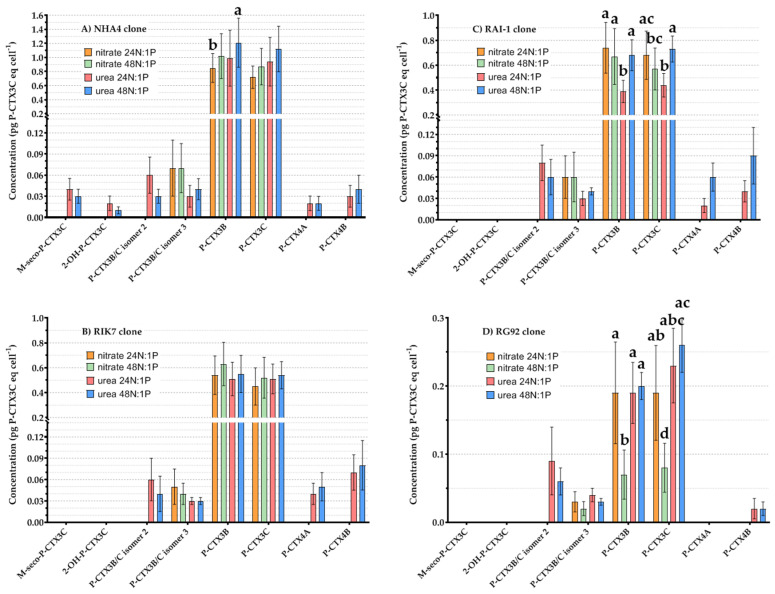
Mean cellular concentrations (expressed in pg P-CTX3C eq cell^−1^) of P-CTX analogs measured by LC-MS/MS in NHA4 (**A**), RIK7 (**B**), RAI-1 (**C**), and RG92 (**D**) clones grown in nitrate 24N:1P and 48N:1P vs. urea 24N:1P and 48N:1P. Data represent the mean ± 95% CI of n = 18 samples (two technical replicates analyzed at three culture ages and three pH conditions). Letters indicate statistical differences observed between samples based on pairwise comparisons.

The variations in P-CTX analogs diversity observed between four strains of *G. polynesiensis* when grown under different N sources and N:P ratios are presented in Figure 5 In nitrate-based experiments, only three quantifiable analogs were detected in cultures, i.e., P-CTX3B/C isomer 3, P-CTX3B and -3C with no significant effects of N:P ratio on their respective levels, except for clone RG92 (Figure 5D). In urea-based experiments, the number of quantifiable analogs substantially increased to five analogs in RG92 (Figure 5D), six in RAI-1 and RIK7 (Figure 5B,C), and eight in NHA4 (Figure 5A).

#### 2.4.4. Effects of pH on *G. polynesiensis* P-CTX Concentrations

**Figure 6:**
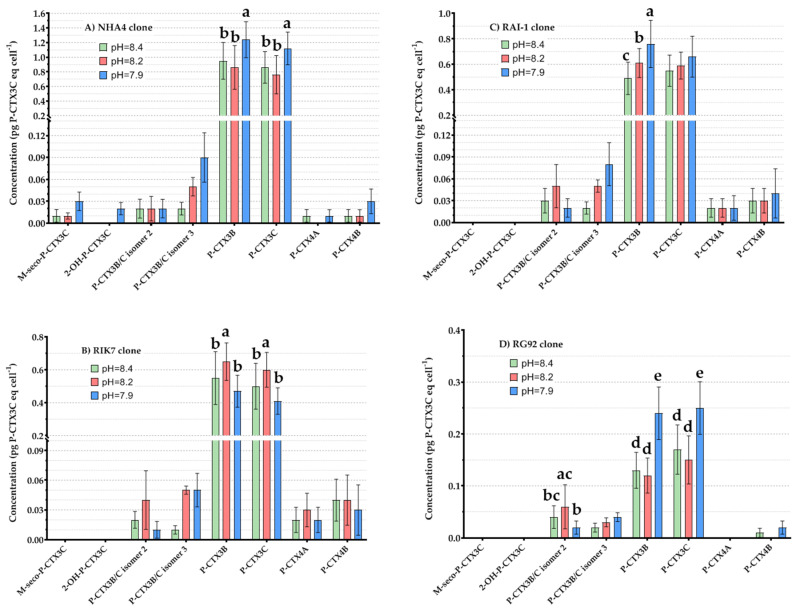
Mean cellular concentrations (expressed in pg P-CTX3C eq cell^−1^) of P-CTX analogs measured by LC-MS/MS in NHA4 (**A**), RIK7 (**B**), RAI-1 (**C**), and RG92 (**D**) clones under three different pH conditions. Data represent the mean ± 95% CI of n = 24 samples (two technical replicates analyzed at three culture ages and two different N sources tested at two N:P ratios). Letters indicate statistical differences observed between samples based on pairwise comparisons.

The variations in P-CTX analogs diversity observed in strains of *G. polynesiensis* when grown under different pH conditions are illustrated in Figure 6. It can be seen that in RAI-1, amounts of P-CTX3B increased significantly from pH = 8.4 to pH = 7.9 (Figure 6C). In NHA4 and RG92, amounts of P-CTX3B and -3C did not vary significantly at pH = 8.2 and pH = 8.4 but were significantly higher at pH = 7.9 (Figure 6A,D). In RIK7, amounts of P-CTX3B and -3C were significantly higher at pH = 8.2 (Figure 6B). In addition, the levels of a minor, more polar analog, namely P-CTX3B/C isomer 3, tended to increase at pH = 7.9 in all the clones as compared to 8.4 (Figure 6A–D). The same observations apply to two other analogs, i.e., 2-OH-P-CTX3C and M-seco-P-CTX3C, which were detected at quantifiable amounts only in NHA4 (Figure 6A). In contrast, P-CTX3B/C isomer 2 and P-CTX4A/4B amounts remained stable across all pH values (Figure 6), except for P-CTX3B/C isomer 2 in RG92, which was significantly higher in pH = 8.2 (Figure 6D).

Table 3 summarizes the different parameters showing significant positive or negative effects on cell yields, overall toxin content, and/or individual analog concentrations in *G. polynesiensis* strains, based on multiple statistical comparisons. Results indicate that the clonal strain influenced both the diversity (number of analogs) and concentrations of each P-CTX analog produced. The final cell yield, the overall cell toxin content, and the levels of P-CTX analogs with low polarity significantly increased in aged cultures, whereas growth stage had no significant effect on the amounts of more polar analogs, except for P-CTX3B/C isomer 2. Contrastingly, decreasing the pH in the culture medium led to reduced cell yields and stimulated the production of more polar forms of P-CTX analogs, while no significant effect was noticed on less polar analogs, except for P-CTX3B. Overall, a 2-fold increase in nitrogen concentrations (from 24N:1P to 48N:1P) resulted in enhanced cell biomass but had little effect (if any) on the overall cell toxin content and the amounts of the various P-CTX analogs composing *G. polynesiensis* toxin profile. Finally, modifying the nitrogen source from nitrate to urea significantly reduced the final cell yields but was highly beneficial to the production of several P-CTX analogs.

## 3. Discussion

Ciguatera occurrences at a global scale depend on a combination of trend drivers. Among them, global ocean warming attributable to global change is often cited as a main driver of the current expansion of CP to novel areas and reportedly increased CP incidence (see References [23,24] for reviews and references therein). In contrast, the potential impacts of ocean acidification and eutrophication on CP trends is a topic that remains largely under-studied. The present work aimed at assessing the growth and toxin production in four toxic clones of *G. polynesiensis* isolated from French Polynesia when grown under different combinations of pH, nitrogen ratios, and sources, three environmental parameters regarded as representative of ocean acidification and eutrophication. Several experimental constraints were encountered in the course of this study, such as the limited number of technical replicates set up in growth experiments, or the lack of replicate measures and certified reference standards in LC-MS/MS analyses. Also, the direct addition of HCl drops in *G. polynesiensis* culture medium for pH stabilization appears as a less ecologically relevant method than bubbling with CO_2_-enriched air. Hence, the data presented here should be regarded as preliminary results pending further confirmatory studies.

Despite these limits, this study yielded interesting novel results. In particular, it was shown for the first time that when grown at pH = 7.9, a value close to the predicted ocean pH for the end of this century, cells of *G. polynesiensis* were able to survive, even if they required higher pH to achieve optimal growth. Cellular processes (such as phytoplankton productivity and toxin production) have been shown to be sensitive to shifts in CO_2_ concentrations, including in diatoms and dinoflagellates [69,70,71,72], but there is still a striking lack of published data on the response of *Gambierdiscus* spp. to varying pH. To our knowledge, only one other study has examined the effects of pH on the growth and toxicity of two strains isolated from Marakei Island (Republic of Kiribati) and identified as *G. balechii* [73] and *Gambierdiscus* sp. type 5 [74] (*Chan, pers. comm*.). In that study, low pH values stimulated the growth while negatively impacting the toxin production in *Gambierdiscus* sp. type 5, which is in marked contrast with our results on *G. polynesiensis*. These contrasting findings suggest that *Gambierdiscus* growth as a response to acidification might differ across species, and that ocean acidification may result in a shift in both the composition and abundance of *Gambierdiscus* spp. in the environment. Regarding the effect of pH on *G. polynesiensis* toxin production, this study showed that acidification of the culture medium stimulated the production of more polar (oxidized) forms of P-CTXs in this species, i.e., P-CTX3B/C isomer 3 in all four clones, and 2-OH-P-CTX3C and M-seco-P-CTX3C in NHA4. It is well established that the biooxidation undergone by CTXs in the food chain leads to more toxic analogs [2]. Our findings thus suggest that ocean acidification may result in less abundant but also more toxic populations of *G. polynesiensis*, a major contributor to toxin flux in ciguateric biotopes in the Pacific [30,33,68,75,76], which, in turn, may lead to increased CP risk. Indeed, it is recognized that the toxicity in a given area depends primarily on the presence of selected highly toxic species of *Gambierdiscus* that may not be the numerically dominant species, but which contribute disproportionately to the overall toxicity of the region [33,77]. In any case, since ciguatera occurrence is generally associated with complex assemblages of multiple *Gambierdiscus* species [15,68,78], these preliminary observations warrant further studies on the physiological and toxicological responses of the other *Gambierdiscus* species frequently encountered in benthic assemblages of ciguateric biotopes, in order to inform reliable risk models on CP trends in the context of global change.

The role played by nutrients in ciguatera events remains unclear. Again, very few field studies with reported statistical results have measured nutrient concentrations concurrently to ciguatera dinoflagellate densities. Of these, both Carlson and Tindall [62] and Loeffler et al. [60] reported a positive correlation between nutrient concentrations and *Gambierdiscus* abundance in the U.S. Virgin Islands. Conversely, Yasumoto et al. [57] and Ichinotsubo et al. [59] found no direct correlation between nutrients and cell densities in French Polynesia and Hawaii respectively, consistent with the observations by Parsons and Preskitt [58] in several Hawaiian locations, although according to these authors, the highest cell densities occurred at the site with the highest nutrient concentrations. All this data suggest that nutrient loadings in coastal environments may not necessarily lead to *Gambierdiscus* blooms and, in fact, the linkage between increased HAB occurrences and eutrophication remains largely inconclusive for a number of microalgal taxa (References [63,78,79] and references therein). However, according to Skinner et al. [80], nutrient inputs can cause a major shift in the distribution and abundance of ciguatera-related dinoflagellates, as evidenced in the Northern Great Barrier Reef (Australia): in several locations affected by on-going coastal eutrophication, benthic assemblages in inshore reefs once dominated by *Gambierdiscus* were composed primarily of *Prorocentrum* and *Ostreopsis*, whilst *Gambierdiscus* was still dominant in offshore locations.

In the present study, the influence of nitrogen (nutrient levels and nitrogen sources) on the growth and toxicity of *G. polynesiensis* was examined in in vitro conditions. Nitrogen was selected because French Polynesian lagoons are usually characterized by low concentrations of dissolved mineral nitrogen [47,48,49]. Our results indicate that growth rates did not significantly vary with N:P ratio, consistent with the findings of Sperr and Doucette [67], who tested a wide range of N:P ratios from 5:1 to 50:1 on several Caribbean strains. Another main finding was that all four *G. polynesiensis* strains achieved higher growth rates with nitrate rather than with urea. Likewise, the cell biomass yielded at D30 were 3-fold higher on average in nitrate than in urea at 24N:1P for all four strains. These results substantiate previous observations by Lartigue et al. [32], who tested the effects of different nitrogen sources on the growth and toxicity of two *Gambierdiscus* strains subsequently classified as *G. caribaeus* and *Gambierdiscus* ribotype 2 [8,77]. They noted that both strains grew well when the nitrogen source was nitrate while neither species grew on urea. These findings are in marked contrast with those of Durand-Clement [65,66], who reported increased growth in *Gambierdiscus* when urea concentration was increased from 0 to 1 mM. Parsons et al. [27] hypothesized that one possible explanation for this apparent contradiction is that Durand-Clément [66] tested a different species of *Gambierdiscus,* thereby implying that the various *Gambierdiscus* species likely have different nutrient physiologies.

The influence of nutrients in stimulating *Gambierdiscus* toxin production is also poorly documented and remains ambiguous. The field study conducted by Okolodkov et al. [61] in the Yucatan peninsula (Gulf of Mexico) outlined the importance of potential linked effects of various environmental parameters as the authors hypothesized that the greatest potential for ciguatoxin flux into the food web may occur in protected, low turbulence environments, where salinities are high, nutrients abundant, and water temperatures are between 24 and 31 °C.

In cultured-based studies, Lechat et al. [64] showed that variations in media (use of media with a high metallic salt content) resulted in increased ciguatoxin production, whereas Lartigue et al. [32] claimed that CTX production in Caribbean *G. toxicus* clones was not affected by organic (nitrate or ammonium) or inorganic (urea, free amino acids or putrescine) nitrogen sources. In the present study, we were able to confirm that the use of either nitrate or urea in the growth medium had no significant effect on the overall toxin yield in *G. polynesiensis* cultures, but did result in a substantial increase of quantifiable analogs, from three to a maximum of eight P-CTX analogs depending on the strain considered. The reason for this drastic change in *G. polynesiensis* toxin profile following the shift to urea in the culture medium is unclear. Epiphytic/benthic dinoflagellates have classical nitrogen requirements: they are known to readily utilize nitrate and ammonium with some taxa showing an apparent preference for the latter [81,82]. Both Bomber [83] and Lartigue et al. [32] found an increase in cellular ciguatoxicity of *Gambierdiscus* with increasing NH_4_^+^, which might indicate a functional role of this compound in the biosynthesis of CTXs. However, the potential role played by intracellular phosphorus utilization in toxin synthesis should not be overlooked [84,85]. Concerning the influence of N:P ratio on toxin production, Durand-Clement [65] indicated that overall toxin production remained constant with nutrient levels, whereas Sperr and Doucette [67] concluded that CTX production can be determined by high N:P ratio (i.e., N:P ≥ 30:1) in Caribbean strains of *G. toxicus*.

It should be noted that many of these earlier studies have not evaluated toxicity with CTX-specific protocols and that none of these studies have evaluated individual toxins in the response of *Gambierdiscus* clones to different nutrient regimes. Indeed, quantitation methods used in studies by Durand-Clement [65] and Sperr and Doucette [67] were based on an overall toxins concentration estimate (i.e., crude extract evaluated by mouse bioassay) which is not directly comparable with our use of LC-MS/MS method in the present study, which is more specific for P-CTX detection and in which each known P-CTX analog concentration is investigated. Another issue which makes comparisons with previous studies difficult is that much of the early work with *Gambierdiscus* referred solely to *G. toxicus*, while it is now well established that multiple species exist within this genus.

Dinoflagellates in the genus *Gambierdiscus* are the source of a number of non-structurally related groups of secondary metabolites of interest (see Reference [20] for a review and references therein). Of these, CTXs are by far the most studied group due to their direct involvement in ciguatera poisoning. At present, only P-CTX3C is commercially available for detection and quantitation purposes. The current scarcity of CTX analytical standards constitutes a serious hindrance to the quantitation of these compounds in seafood and the implementation of food safety surveillance programs. Hence, significant efforts have been devoted in the past decade to determine which species of *Gambierdiscus* are more toxic than the others, and assess the variations in toxin production in response to different stressors (see References [27,28] for reviews and references therein). Not all species of *Gambierdiscus* are known to produce CTXs. Among the eighteen species described to date, *G. polynesiensis*—a species allegedly endemic to the Pacific Ocean—is recognized as the highest CTX producer with published toxicity data ranging from 1.2 up to 20.9 pg P-CTX3C eq cell^−1^ [17,19,30,33,86]. In the present study, we showed that *G. polynesiensis* cellular toxicity could vary by up to 46 orders of magnitude in French Polynesian clones from geographically distinct origins. Although part of these variations was attributable to different culture conditions and growth stage, these observations highlight the fact that an adequate selection of the appropriate candidate-strains is preponderant in R&D programs aiming at the custom production of high-value CTX standards. Of note, this strain-dependent production of CTXs demonstrated in *G. polynesiensis* is also well established in other species such as *G. australes*, *G. balechii*, *G. pacificus*, *G. toxicus*, *F. paulensis,* and *F. ruetzleri* [3,9,15,74,75,87,88]. In addition, numerous studies have demonstrated that cellular toxin levels often increase with the age of the culture [17,29,32,33,67], implying that the culture conditions for optimal growth of *Gambierdiscus* are likely to differ from the ones ensuring optimal toxin production. The LC-MS/MS data presented here (and those of Longo et al. [33]) also revealed that an increase in the number of CTXs analogs produced in cultures generally takes place from the exponential to the stationary phase. Finally, another important issue that needs to be considered in toxin production programs is the gradual drop in toxin yields likely to affect high toxin-producing clones over time, as already reported in *G. polynesiensis* clones isolated from the Cook Islands and French Polynesia, respectively [33,89]. These observations suggest that high toxin production may not be a stable characteristic in long-term cultures of *G. polynesiensis*, contradicting earlier observations by Chinain et al. [17].

## 4. Conclusions

This study has provided clear evidence that manipulation of environmental variables can result in enhanced growth and toxicity (types of P-CTX analogs in addition to amounts) in *G. polynesiensis*. However, the importance of other factors such as culture age, clonal differences, and harvest time should not be overlooked if the pursued objective is to maximize toxin yield and diversity. More widely, increasing attention is paid to the secondary metabolites produced by microalgae, particularly dinoflagellates, due to their potential uses in the biological, biomedical, and toxicological fields [90]. *Gambierdiscus* is no exception to the rule. Further studies are needed on the other species of *Gambierdiscus* also regarded as major contributors to toxin flux in CP-prone areas, e.g., *G. excentricus, G. australes,* and possibly *G. silvae* [14,16,91], thereby providing important information that can greatly benefit current efforts to establish a sustainable source of CTX standards. Indeed, numerous studies have shown the ability of *Gambierdiscus* to produce a wide variety of P-CTX analogs as well as their bioaccumulation at each trophic level, from herbivorous to carnivorous fish or marine invertebrates [28,92]. However, despite the identification of several C-CTX and I-CTX analogs in carnivorous fish and sharks [93,94,95,96,97,98,99,100], no study has yet been able to demonstrate the toxin production of C-CTXs or I-CTXs in *Gambierdiscus*. This fact is of major importance as toxin-producing in vitro cultures can provide a continuous source of CTX standards. Easier access to a variety of CTX standards will undoubtedly help improve the testing capabilities in ciguatera management and hence, accelerate the implementation of food safety surveillance programs globally. In this process, studies on the complementarity between several detection methods classically used in CP risk monitoring program (e.g., LC-MS/MS, CBA-N2a, and Receptor Binding Assay) need to be pursued [92,101,102,103] to better understand quantitation differences across methods and laboratories, and hence foster trans-regional comparative studies on ciguatera. Concurrently, the recent development of metabolomics appears as a unique opportunity to conduct in-depth investigations on the chemodiversity of these dinoflagellates, in order to, e.g., describe new compounds (including toxins), assess the variations of their productions in response to different stressors, or identify chemical markers at different taxonomic levels [103].

## 5. Materials and Methods

### 5.1. Source of G. polynesiensis Isolates

The four clones of *G. polynesiensis* used in this study (RIK7, NHA4, RAI-1, and RG92), originate from different islands of French Polynesia, i.e., Mangareva Island (Gambier archipelago), Nuku Hiva Island (Marquesas archipelago), Raivavae Island (Australes archipelago), and Rangiroa atoll (Tuamotu archipelago) (Table 4). These clones were isolated from macro-algal and/or artificial substrate (window screens) samples collected following the methods described by Chinain et al. [17] and Tester et al. [104], respectively. All four strains are part of the Laboratory of Marine Biotoxins culture collection at the *Institut Louis Malardé* (Tahiti, French Polynesia), where cultures are deposited.

### 5.2. In Vitro Cultures

All four *G. polynesiensis* clones selected for this study had been maintained in natural seawater (pH = 8.4 on average) supplemented with f_10k_ medium [105], at 26 ± 1 °C under 60 ± 10 μmol photons m^−2^ s^−1^ irradiance (12 h light:12 h dark photoperiod) for years prior to experimentation. When culture conditions were changed, clones were acclimated for 40 days to the ambient regime prior to growth experiments.

Six consecutive experiments were conducted, three using nitrate as N source at three distinct pH, and the other three, using urea (Sigma-Aldrich, St. Louis, MO, USA). For each experiment at a given pH, the four strains were tested under two N:P ratios and at three culture ages in two technical replicates.

Regarding the two N:P ratios tested, i.e., N:P of 24 and 48, the composition of the f_10k_ medium was modified accordingly. Practically, N and P concentrations were 0.05 µM and 2.1 × 10^−3^ µM in 24N:1P experiments respectively, which are consistent with concentrations observed in oligotrophic ocean water [106]. In 48N:1P experiments, concentrations used for N and P elements were 0.1 µM and 2.1 × 10^−3^ µM, respectively. Even though the N:P ratio 24 is significantly higher than the Redfield ratio (16), it was used as the standard condition since it has been used for many years in the maintenance of cultures at Institut Louis Malardé. It was also verified to be locally found in the Polynesian coastal environment [107,108] and to be comprised between the median N:P ratio measured in warm oligotrophic mid-latitude oceans, i.e., 28N:1P [109], and in global ocean water, i.e., 22N:1P [106].

In pH experiments, the pH in culture flasks was monitored twice a day and stabilized at three different values, i.e., 8.4, 8.2, and 7.9, by direct addition of a few drops of 1 M HCl into the culture medium. Overnight, a +0.3 pH unit variation was observed on average and daily corrected.

Overall, different combinations of culture conditions under varying pH, N:P ratio, and nitrogen sources (N = 144 combinations) were thus tested on *G. polynesiensis* strains. To this end, batch cultures of each of the four clones were established in technical duplicates by inoculating 40,000 cells into 200 mL of seawater supplemented with 200 μL of f_10k_ in 250 mL Erlenmeyer flasks. Seawater was previously aged for 1 month, filtered through a 0.2 μm GF/F Whatman filters (Dutscher, Brumath, France), and sterilized by autoclaving for 20 mn at 120 °C. All cultures were grown in an incubator (ThermoStable IR250, Wigel, Eichel, Germany). Flasks were incubated at randomly determined sites in the incubator and flask position was changed on a daily basis.

### 5.3. Growth Rate

Growth rate was assessed using in vivo fluorescence following the method described in Longo et al. [33]. Briefly, cell growth was assessed by measuring chlorophyll fluorescence at 485 nm, at two-day intervals, using a spectrophotometer (Victor X2, PerkinElmer, Waltham, MA, USA). To limit error during fluorescence measurements, cultures were fully mixed prior to fluorescence reading, then a 100 µL aliquot of each culture was deposited in 96-well plates, where cells were lysed by the addition of 100 µL dimethyl sulfoxide (DMSO; n = 3 wells per batch culture). Five fluorescence readings were performed on each well.

Growth rates (µ) were determined using the following Equation (1):

µ = [ln*(rfu_t1_/rfu_t0_)]/[ln (2) * (t_1_ − t_0_)] (1)

in which μ (division day^−1^) is the growth rate, and rfu_t1_ and rfu_t0_ represent the fluorescence measured at times t_1_ (days) and t_0_ respectively, corresponding to the exponential growth phase portion of the growth curve.

### 5.4. Cells Harvest and Toxin Extraction

Cells were harvested at D30 post-inoculation. Prior to cell harvest, the total cell yield was determined by automated counting using a Multisizer III^TM^ particle counter (Beckman Coulter, Inc., Brea, CA, USA). Cultures were harvested by filtration onto a 40 µm sieve in their late exponential, early stationary, and senescence growth phase (i.e., at D10, D21, and D30 post-inoculation, respectively). Each filter was then transferred into a 50 mL Greiner tube using seawater, and centrifuged at 2800× *g* for 2 min. After discarding the supernatants, filters were freeze-dried for 20 h at −20 °C, 1 mbar, then 4 h at −60 °C, 0.01 mbar (Martin Christ, Beta 1–8 LDplus, Osterode am Harz, Germany). Each sample was then stored at −20 °C until further extraction for toxicity screening.

*G. polynesiensis* cells were extracted by adding 20 mL of methanol (MeOH) directly into the tubes containing each freeze-dried cell sample. Tubes were then vortexed for a few seconds and then placed in an ultrasound tank for one hour. Following a centrifugation step at 2800× *g* for 10 min, the resulting supernatant was recovered in a 250 mL flask. This extraction step was repeated once in MeOH and then once in aqueous methanol (MeOH: H_2_O 1:1). The three supernatants were further pooled and dried under vacuum using a rotary evaporator (Rotavapor RII, Büchi, Flawil, Switzerland), and the resulting crude cellular extract suspended in 4 mL of pure methanol.

Half of this solution (2 mL MeOH) was filtered through a Sartorius filter (3 cm diameter, 0.45 μm porosity), then evaporated under nitrogen to dryness to obtain a crude extract destined for the LC-MS/MS analysis. In total, 288 samples have been generated and analyzed.

### 5.5. Liquid Chromatography Coupled with Tandem Mass Spectrometry (LC-MS/MS) Analysis

The toxin screening of *G. polynesiensis* cell extracts was performed using a Ultra High Performance Liquid Chromatography (UHPLC) system (UFLC Nexera, SHIMADZU, Kyoto, Japan) coupled to a hybrid triple quadrupole-linear ion-trap API4000 QTRAP mass spectrometer (SCIEX, Redwood City, CA, USA) equipped with a TurboV^®^ electrospray ionization source (ESI). The instrument control, data processing, and analysis were conducted using Analyst software 1.6.2 (Sciex, CA, USA). A linear gradient using water as eluent A and MeOH as eluent B, both eluents containing 2 mM ammonium formate and 50 mM formic acid, was run on a Zorbax Eclipse Plus C18 column, 50*2.1 mm, 1.8 μm, 95 Å (Agilent Technologies, Santa Clara, CA, USA). The flow rate was 0.4 mL min^−1^, the injection volume was 5 μL, and the column temperature 40 °C.

The elution gradient was as follows: 78% B to 88% B from 0 to 10 min, hold at 88% B for 4 min, decrease from 88% to 78% in 1 min, and hold for 5 min at 78% B. Mass spectrometric detection was performed in positive ionization mode using Multiple Reaction Monitoring (MRM) scanning.

The optimized ESI+ parameters were set as follows: curtain gas at 25 pounds per square inch (psi), ion spray at 5500 V, turbo gas temperature at 300 °C, gas 1 and 2 were set at 40 and 60 psi respectively, and an entrance potential at 10 V.

MRM acquisition method was created using the scheduled MRM algorithm. This algorithm optimizes dwell times and cycle time to provide better peak detection and improve reproducibility. A detection window of 90 s and a target scan time of 2 s were chosen for the MRM method. In order to quantify P-CTX analog, a calibration solution of P-CTX3C (Wako, Japan) was prepared in MeOH, with concentration ranging from 12 to 200 ng mL^−1^. LOQ is determined to 10 ng mL^−1^ and LOD to 5 ng mL^−1^. Due to the lack of analytical standards, contents of each P-CTX analog were quantified from the P-CTX3C calibration curve prepared, assuming equivalent molar response. The sum of the estimated concentrations of all analogs allowed characterization of the respective toxin profiles and calculation of the overall cellular toxin content (expressed in pg P-CTX3C eq cell^−1^).

### 5.6. Statistical Analyses

To determine whether growth rates and toxin profiles differences observed between the different culture conditions tested (i.e., different combinations of pH and N:P ratio values as well as the use of different nitrogen sources in the growth medium) were statistically significant, standard deviations (SD) and 95% confidence interval (CI; *p*-value < 0.05) were calculated. Then, an analysis of variance (ANOVA) (normality, homoscedasticity) was performed using RStudio v1.0.153 (Boston, MA, USA) or GraphPad Prism v8.1.2. (GraphPad, San Diego, CA, USA). In order to ensure more robust analyses about the general effects on toxin production, data were merged to increase sample size and allow parametric analysis, even if normality sometimes was not verified. By way of example, to study the effect of geographic origin on P-CTX amounts, results obtained for the 288 samples were grouped into 4 datasets (with n = 72 per dataset) corresponding to the four *G. polynesiensis* clones, regardless of the pH, cell age, and N levels and source. A similar rationale was applied to assess the effects of pH (n = 24), culture age (n = 24), N:P ratio and N source (n = 18) on P-CTX levels of each clone.

## Figures and Tables

**Table 1:** Mean growth rates ± standard deviation (SD) (in div day^−1^, *n* = 2) of *G. polynesiensis* clones (NHA4, RAI-1, RIK7, and RG92) as assessed at day 10 (D10) and day 21 (D21), under three different pH conditions (8.4, 8.2, and 7.9), using either nitrate or urea as nitrogen (N) source when tested at two distinct N:P ratios (24N:1P and 48N:1P).

Culture Age	N Source	N:P Ratio	PH	NHA4	RAI-1	RIK7	RG92
D0→D10	Nitrate	24N:1P	8.4	0.20 ± 0.01	0.18 ± 0.00	0.20 ± 0.03	0.15 ± 0.00
			8.2	0.17 ± 0.01	0.20 ± 0.02	0.17 ± 0.00	0.21 ± 0.01
			7.9	0.22 ± 0.01	0.23 ± 0.00	0.22 ± 0.00	0.18 ± 0.00
			8.4	0.23 ± 0.00	0.15 ± 0.00	0.22 ± 0.02	0.19 ± 0.00
		48N:1P	8.2	0.22 ± 0.01	0.20 ± 0.03	0.21 ± 0.03	0.13 ± 0.00
			7.9	0.26 ± 0.01	0.12 ± 0.01	0.16 ± 0.00	0.21 ± 0.01
	Urea	24N:1P	8.4	0.04 ± 0.01	0.03 ± 0.02	0.08 ± 0.01	0.03 ± 0.02
			8.2	−0.08 ± 0.01	−0.06 ± 0.03	−0.07 ± 0.00	−0.11 ± 0.07
			7.9	−0.05 ± 0.01	−0.05 ± 0.01	−0.04 ± 0.03	−0.05 ± 0.01
		48N:1P	8.4	0.12 ± 0.02	0.11 ± 0.01	0.14 ± 0.01	0.04 ± 0.01
			8.2	0.02 ± 0.03	−0.06 ± 0.01	0.00 ± 0.02	0.00 ± 0.02
			7.9	0.01 ± 0.01	−0.01 ± 0.01	0.00 ± 0.01	−0.02 ± 0.00
D10→D21	Nitrate	24N:1P	8.4	0.09 ± 0.02	0.09 ± 0.00	0.08 ± 0.01	0.13 ± 0.00
			8.2	0.15 ± 0.00	0.07 ± 0.03	0.13 ± 0.01	0.09 ± 0.01
			7.9	−0.02 ± 0.02	−0.06 ± 0.04	0.00 ± 0.01	−0.07 ± 0.03
		48N:1P	8.4	0.08 ± 0.01	0.13 ± 0.01	0.08 ± 0.03	0.11 ± 0.00
			8.2	0.07 ± 0.04	0.04 ± 0.04	0.08 ± 0.02	0.15 ± 0.00
			7.9	0.04 ± 0.08	−0.05 ± 0.02	0.00 ± 0.00	−0.07 ± 0.01
	Urea	24N:1P	8.4	0.05 ± 0.01	0.05 ± 0.02	0.07 ± 0.05	0.05 ± 0.01
			8.2	0.15 ± 0.02	0.11 ± 0.01	0.16 ± 0.01	0.17 ± 0.05
			7.9	0.04 ± 0.02	0.07 ± 0.02	0.06 ± 0.07	0.02 ± 0.01
		48N:1P	8.4	0.07 ± 0.01	0.10 ± 0.01	0.09 ± 0.02	0.10 ± 0.03
			8.2	0.17 ± 0.03	0.16 ± 0.04	0.20 ± 0.03	0.17 ± 0.01
			7.9	0.09 ± 0.01	0.11 ± 0.00	0.13 ± 0.01	0.10 ± 0.01

**Table 2:** Mean ± SD (n = 2) of final cell biomass yielded at D30 for each *G. polynesiensis* clones (NHA4, RAI-1, RIK7. and RG92), under three different pH conditions (8.4, 8.2, and 7.9), and two distinct N:P ratios (24N:1P and 48N:1P) when nitrate or urea were used as N source.

N Source	N:P Ratio	PH	NHA4	RAI-1	RIK7	RG92
Nitrate	24N:1P	8.4	359,210 ± 62,793	235,365 ± 42,083	327,392 ± 27,340	355,867 ± 10,714
		8.2	369,212 ± 16,423	187,680 ± 13,356	351,475 ± 36,808	224,060 ± 33,932
		7.9	130,333 ± 11,857	151,357 ± 17,596	166,912 ± 64,067	171,303 ± 38,440
	48N:1P	8.4	295,035 ± 49,244	222,077 ± 39,746	264,010 ± 49,939	412,392 ± 19,155
		8.2	386,863 ± 69,359	165,353 ± 24,543	299,937 ± 7,630	203,235 ± 17,276
		7.9	189,182 ± 12,229	122,088 ± 23,412	238,283 ± 14,594	169,433 ± 16,245
Urea	24N:1P	8.4	95,207 ± 1,202	102,435 ± 27,400	198,646 ± 48,861	108,437 ± 44,900
		8.2	87,269 ± 21,779	101,969 ± 11,809	100,867 ± 21,885	94,374 ± 2,404
		7.9	59,397 ± 1,465	75,607 ± 17,721	67,019 ± 9,035	55,444 ± 11,268
	48N:1P	8.4	283,196 ± 81,070	259,798 ± 14,566	256,197 ± 8,309	120,589 ± 38,608
		8.2	245,882 ± 23,122	212,219 ± 27,011	249,582 ± 37,724	243,359 ± 13,188
		7.9	125,726 ± 15,384	175,371 ± 18,907	164,946 ± 6,384	110,912 ± 15,593

**Table 3:** Effects of strain, culture age, N:P ratio, N source, and pH on the cell biomass, overall toxin content, and P-CTXs profile in *G. polynesiensis*. The statistical significance of each of these parameters is indicated. The different P-CTX analogs are listed by increasing polarity. The “→ “symbol indicates which condition comparison has been conducted.

Parameter	Strain	Culture Age D10→D21/D30	N:P Ratio 24N:1P→48N:1P	N Source Nitrate→Urea	pH 8.4→8.2/7.9
Cell yields at D30	ns	⭧ ***	⭧ *	⭨ ***	⭨ **
Overall toxin content	***	⭧ *	ns	ns	ns
P-CTX4A	***	⭧ ***	⭧ *	⭧ ***	ns
P-CTX4B	***	⭧ ***	⭧ **	⭧ ***	ns
P-CTX3B	***	⭧ ***	ns	ns	⭧ *
P-CTX3C	***	⭧ ***	ns	⭧ *	ns
P-CTX3B/C isomer 2	*	⭨ ***	⭨ *	⭧ ***	⭧ ***
P-CTX3B/C isomer 3	***	ns	ns	⭨ ***	⭧ ***
2-OH-P-CTX3C	***	ns	ns	⭧ ***	⭧ **
M-seco-P-CTX3C	***	ns	ns	⭧ ***	⭧ **

* *p*-value < 0.05, ** *p*-value < 0.01, *** *p*-value < 0.001, ns = non significant, ⭧ increase, ⭨ decrease.

**Table 4:** Geographic origin and year of isolation of the four *G. polynesiensis* clones selected for this study.

ID Code	RIK7	NHA4	RAI-1	RG92
Year of isolation Archipelago	2013 Gambier	2015 Marquesas	2008 Australes	1992 Tuamotu
Island	Mangareva	Nuku Hiva	Raivavae	Rangiroa

## Supplementary Materials

The following are available online at https://www.mdpi.com/2072-6651/12/12/767/s1, Table S1. List of all *m*/*z* transitions required in scheduled MRM LC-MS/MS analysis to detect and quantify P-CTX compounds.
