# Supplementary material for: Effects of pH and Nutrients (Nitrogen) on Growth and Toxin Profile of the Ciguatera-Causing Dinoflagellate Gambierdiscus polynesiensis (Dinophyceae)
Source: Toxins (Basel). 2020 Dec 4;12(12):767. doi: 10.3390/toxins12120767 (PMC7761829; doi:10.3390/toxins12120767)
Supplement: Supplementary file 1 [file toxins-12-00767-s001.pdf]

Supplementary Material: Effects of pH and Nutrients (Nitrogen) on Growth and Toxin Profile of the Ciguatera-Causing Dinoflagellate *Gambierdiscus polynesiensis* (Dinophyceae)

Sébastien Longo, Manoëlla Sibat, Hélène Taiana Darius, Philipp Hess and Mireille Chinain

Table S1. List of all *m/z* transitions required in scheduled MRM LC-MS/MS analysis to detect and quantify P-CTX compounds.

COMPOUND	PARENT ION SPECIES	*m/z* TRANSITION	CORRESPONDING COLOR
P-CTX3B, P-CTX3C and isomer (4)	$[M+NH_4]^+$	1040.6 → 1005.6	black
	$[M+H]^+$	1023.6 → 1005.6	dark purple
		125.1	flashy green
P-CTX3B/C isomers group (1) (2) (3)	$[M+NH_4]^+$	1040.6 → 1005.6	purple
	$[M+H]^+$	1023.6 → 1005.6	red
		125.1	turquoise
P-CTX4A and P-CTX4B	$[M+NH_4]^+$	1078.6 → 1043.6	dark blue
	$[M+H]^+$	1061.6 → 1043.6	Bordeaux red
		125.1	khaki
M-seco-P-CTX3C	$[M+H]^+$	1041.6 → 1023.6	black
		1005.6	blue
		125.1	flashy green
2-OH-P-CTX3C	$[M+NH_4]^+$	1058.6 →1023.6	light blue
		1005.6	pink
		125.1	orange
